# Sources of Caffeine in Diets of US Children and Adults: Trends by Beverage Type and Purchase Location

**DOI:** 10.3390/nu8030154

**Published:** 2016-03-10

**Authors:** Adam Drewnowski, Colin D. Rehm

**Affiliations:** Center for Public Health Nutrition, University of Washington, Seattle, WA 98195, USA; colin.rehm@gmail.com

**Keywords:** caffeine, beverages, children, NHANES, trends

## Abstract

New sources of caffeine, besides coffee and tea, have been introduced into the US food supply. Data on caffeine consumption age and purchase location can help guide public health policy. National Health and Nutrition Examination Surveys (NHANES) were used to estimate population-level caffeine intakes, using data from 24-h dietary recall. First, caffeine intakes by age-group and beverage type were estimated using the most recent 2011–2012 data (*n* = 7456). Second, fourteen years trends in caffeine consumption, overall and by beverage type, were evaluated for adults and children. Trend analyses were conducted by age groups. Last, trends in caffeine intakes by purchase location and beverage type were estimated. In 2011–2012, children aged four to eight years consumed the least caffeine (15 mg/day), and adults aged 51–70 years consumed the most (213 mg/day). The population mean (age ≥ four years) was 135 mg/day, driven largely by coffee (90 mg/day), tea (25 mg/day), and soda (21 mg/day). For the 14–19 years and 20–34 years age-groups, energy drinks contributed 6 mg/day (9.9%) and 5 mg/day (4.5%), respectively. The bulk of caffeine came from store-bought coffee and tea. Among both children and adults combined, caffeine intakes declined from 175 mg/day (1999–2000) to 142 mg/day (2011–2012), largely driven by a drop in caffeine from soda (41 mg/day to 21 mg/day). Store-bought coffee and tea remain principal drivers of caffeine intake in the US. Sodas and energy drinks make minor contributions to overall caffeine intakes.

## 1. Introduction

Assessing the dietary sources of caffeine among US children and adults has implications for public health policy. Caffeine, a mild stimulant [1,2], is a frequently consumed dietary constituent, which is an added ingredient or is naturally present in varying amounts in both beverages and solid foods [3,4,5,6,7]. Caffeine is recognized by the US Food and Drug Administration as safe for adults in amounts < 400 mg/day [8]. The 2015 Dietary Guidelines Advisory Committee (DGAC) report concluded that there was only limited evidence to link high-dose caffeine intakes with health risks for children and adults [9]. However, whereas the high dose for adults was set at 400 mg/day, the dose for children was not determined [9]. Some concerns remain about the consumption of energy drinks by children and teenagers, particularly due to their high caffeine content, but also due to their energy content [10,11].

Most dietary caffeine is provided by beverages [3], with foods such as chocolate providing relatively small amounts. Coffee contains more caffeine than do most beverages and the amounts consumed depend on age [3]. Adults drink coffee, but young children generally do not [3,11]. In contrast, caffeinated energy drinks are most often consumed by teenagers and young adults [2,12,13]. The consumption of carbonated sugar sweetened beverages (SSBs) begins in childhood, peaks in young adult life, and declines thereafter [14]. As a result, the chief sources of dietary caffeine show sharp differences by age [3,15]. To complicate matters, age-driven patterns of beverage consumption are subject to secular trends [3]. Whereas the consumption of energy drinks has increased in recent years, the consumption of caffeinated sugar-sweetened beverages has, to the contrary, declined [6,14].

Based on recent analyses of 24-h recalls from the nationally representative NHANES 1999–2010 database, 70% of children and adolescents (aged < 22 years) consumed some caffeine on any given day [11,15]. However, no net increases in caffeine intakes were observed during that time [11,15]. Analyses of seven-day food diaries from the Kantar Worldpanel [3] reported that 85% of the US population aged ≥ two years consumed at least one caffeinated beverage per day. Mean caffeine intake across all age groups was estimated at 165 mg/day, largely driven by coffee consumption.

The present analyses were based on seven consecutive NHANES cycles (1999–2012) with a total of 56,207 respondents aged ≥ four years. First, detailed analyses of caffeine sources by beverage type and age group were based on the most recent 2011–2012 NHANES cycle (*n* = 7456). First, the contribution of different beverages and foods to caffeine consumption for each age group was assessed both in terms of absolute amounts (mg/day) and in terms of percent contribution to total intakes. Second, we examined fourteen-year trends in total caffeine consumption, overall and by beverage type. Lastly, analyses of time trends by purchase location for children and adults were based on NHANES 2003–2012. By including both children and adults, the present analyses complement and extend earlier reports to identify the major sources of caffeine by beverage type and purchase location.

## 2. Methods

### 2.1. Dietary Intake Data

The current study was based on 7 cycles of the nationally representative National Health and Nutrition Examination Survey (NHANES) corresponding to years 1999–2012 [16,17,18,19]. Data from the most recent cycle (2011–2012) for 2655 children (4–19 years) and 4801 adults (≥20 years) were used to characterize caffeine consumption overall and beverage type, using the most current nationally representative dietary data. For analysis of trends from 1999–2000 through 2011–2012, data for 22,276 children and 33,931 adults was used.

Caffeine consumption data were based on the first 24-h dietary recall, conducted in person by trained interviewers [20]. The interview probed for all foods and beverages consumed from midnight-to-midnight in the preceding 24 h. A second-day telephone recall was available; however, consistent with other studies [11,15], this study used the first recall only so that the data were comparable across all survey cycles.

For the NHANES dietary recall, recalls for children aged 4–5 years were completed by a parent/guardian. For children aged 6–11 years, the child was the primary respondent, but the parent was present and able to assist. Children aged 12–19 years were the primary respondents but could be assisted by an adult. The necessary ethics approval for NHANES had been obtained by the National Center for Health Statistics [19] and all data used was publicly available.

Dietary recalls were transformed into energy and nutrient intakes using the US Department of Agriculture Food and Nutrient Database for Dietary Studies (FNDDS) which contains nutrient composition data, including caffeine, for all foods and beverages consumed by participants [21]. FNDDS includes more than 7000 individual foods and beverages, including approximately 50 coffee/coffee drinks, 30 types of tea, and caffeinated and decaffeinated versions of soda. The database is updated every two-years to account for changes in the food supply and reformulation of foods. The depth of FNDDS permits the in-depth disaggregation of foods/beverages.

### 2.2. Classification of Caffeine Sources

For analyses of caffeine consumption by beverage type we adapted the What We Eat in America Food Categories, developed by USDA [22]. Beverages were first classified into 15 broad categories and those that included caffeine-containing beverages were flagged. The revised categories included coffee, tea, soda (*i.e.*, carbonated SSBs or low-calorie beverages), energy drinks, milk and other beverages. The other beverages included alcohol and meal replacement beverages, which contributed small amounts of caffeine, and fruit juices, fruit drinks, sports drinks, vegetable juice, water and flavored waters, which contained no caffeine. Caffeine in milk was from modest amounts of caffeine in chocolate milk. As some caffeine also comes from solid foods, namely products containing cocoa, we created a foods category consisting of all non-beverage items. As the present study focused on dietary sources of caffeine, data from dietary supplements were not included.

### 2.3. Purchase Locations of Origin

The NHANES database includes information on the locations of origin (e.g., where the food was purchased or otherwise obtained). Overall, the primary locations were supermarkets or other food stores, fast food restaurants (FFR, which includes pizza takeout/delivery and takeaway coffee stores), full service restaurants (FSRs), and schools [23]. The other locations of origin included someone else (*i.e.*, gifts), common pot/common source (e.g., shared coffee pot or fruit basket at work, herein referred to as “common pot”), vending machines, other types of cafeterias (e.g., workplace), tavern/bar, or sporting/cultural/entertainment events [20]. The store category did not separate grocery stores, supermarkets, convenience stores, pharmacies and specialty food stores. For this study, the primary purchase locations were limited to grocery stores, FFRs, FSRs, common coffee pot, and a combined “other” category. This information was first collected in the 2003–2004 NHANES cycle, and has been collected in every subsequent cycle, allowing for the evaluation of trends in the origin of caffeine from 2003 to 2012.

### 2.4. Statistical Analysis

Descriptive analyses examined caffeine consumption using NHANES 2011–2012 data, overall, and by age group (4–8 years, 9–13 years, 14–19 years, 20–34 years, 35–49 years, 50–70 years, ≥71 years), sex, race/ethnicity (non-Hispanic white, non-Hispanic black, non-Hispanic Asian, Mexican-American, and other Hispanic), family income-to-poverty ratio (<1.3 (generally eligible for Supplemental Nutrition Assistance Program (SNAP) and Women Infant’s and Children (WIC) program), 1.3–1.849 (eligible for WIC, not SNAP), 1.85–2.99 and ≥3), educational attainment (<high school, high school graduate, some college and college graduate or higher), and employment status (employed *vs.* not employed). Family income-to-poverty ratio represents the ratio of family income to the federal poverty guidelines. For example, in 2012 for a family of four, the poverty threshold was $23,050. Pairwise comparisons using a survey-weighted *t*-test were made comparing caffeine consumption at each variable level to a reference group. Analyses by education and employment status were not conducted for children/adolescents. Using the 2011–2012 data we also examined the absolute and proportional amount of caffeine from seven sources (*i.e.*, coffee, tea, soda, energy drinks, milk, other beverages and foods), overall and by age groups.

Overall trends in caffeine consumption, and by type of beverage, were evaluated for children and adults separately. Additional analyses evaluated heterogeneity in trends by age group, which was evaluated by fitting a model with an interaction term for age group and survey cycle. In the evaluation of overall trends in caffeine consumption, limited sensitivity analyses were conducted by removing the few respondents who consumed >1000 mg/day of caffeine (>10.5 cups of coffee) to determine if extreme values could explain any observed trends. We also evaluated whether changing demographics in the US from 1999 to 2012 could explain caffeine consumption trends, by including age and race/ethnicity as covariates in the linear regression model.

From 2003 to 2004 onward, trends in caffeine consumption by location of origin were evaluated for children and adults, separately. Locations of origin evaluated included stores, fast food restaurants/coffee shops, full-service restaurants, common coffee pot, and other sources. Trends in each source of caffeine were evaluated using a survey-weighted linear regression with survey cycle/year as a continuous variable. Data from five cycles including location of origin data (e.g., 2003–2004 through 2011–2012) were pooled to provide a summary estimate of sources of caffeine over the study period. Additional analyses evaluated trends in caffeine consumption stratified by location of origin (e.g., stores or fast food/coffee restaurant) and type of beverage (e.g., coffee, tea, or soda), with trends evaluated in a similar manner.

All analyses accounted for the complex survey-design of NHANES data and were conducted using Stata 13.1 (College Station, TX, USA, 2013).

## 3. Results

### 3.1. Current Patterns of Caffeine Consumption

Overall caffeine consumption, and caffeine consumption by age, gender, race/ethnicity, family income, education, and employment status in 2011–2012 are shown in Table 1. Children and adolescents consumed 35 mg/day caffeine, whereas adults consumed an average of 173 mg/day. Average intake was 196 mg/day for men and 151 mg/day for women. For both children and adults, caffeine consumption was highest in the non-Hispanic white population and lowest in the non-Hispanic black population. There were no major effects of income, though low-income adults appeared to consume less caffeine than higher-income adults (*p* = 0.029). This difference was driven by the fact that lower-income adults were also younger (*p* = 0.20 after adjusting for age group). There were no systematic effects by education, though individuals with some college education, but no degree, consumed more caffeine than adults with a college degree (age-adjustment did not alter this association). By contrast, higher caffeine intakes were linked to employment. Employed adults consumed significantly more caffeine than adults who were not employed. Adjusting for age and limiting the analysis to those 20–64 years (*i.e.*, working-age adults) did not alter the observed association between higher caffeine consumption among employed compared to non-employed adults.

**Figure 1:**
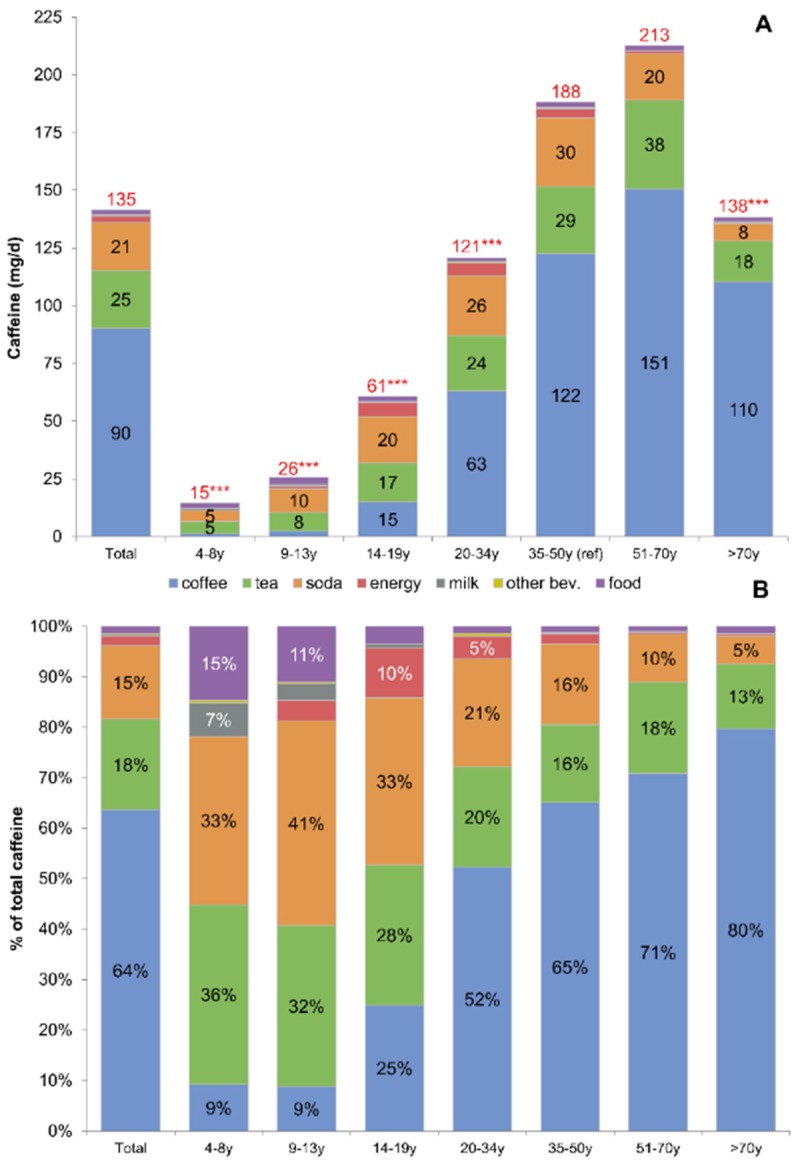
Absolute ((**A**), mg/day) and proportional ^a^ ((**B**), % of total) sources of caffeine from beverages and foods by age group, NHANES 2011–2012. Asterisks represent pairwise survey-weighted *t*-test with age 35–50 years as the reference group; *** *p* < 0.001; ** 0.001 < *p* <0.01; * 0.01 < *p* < 0.05. ^a^ Values less than 5% in Panel B are unlabeled, but include the following. For the total population, values were: energy drinks 2%, flavored milk 0.2%, other beverages 0.2%, and food 1.5%. For age 4–8 years, values were: energy drinks 0% and other beverages 0.5%). For age 9–13 years, values were: energy drinks 4.0%, flavored milk 3.3%, and other beverages 0.3%). For age 14–19 years, values were: flavored milk 0.8%, other beverages 0.1%, and food 3.4%. For age 20–34 years, values were: flavored milk 0.1%, other beverages 0.5%, and food 1.4%., For age 35–49 years, values were: flavored milk 0.1%, other beverages 0.5%, and food 1.4%. For age 51–70 years, values were: energy drinks 0.4%, flavored milk 0.1%, other beverages 0.1%, and food 1.0%. For age > 70 years, values were: energy drinks 0.3%, flavored milk 0.2%, other beverages 0.1%, and food 1.3%.

Figure 1 shows the contribution of different beverages and foods to caffeine consumption among NHANES 2011–2012 participants aged ≥ four years (*n* = 7456). The data are presented overall and separately for each age group, with absolute amounts of caffeine shown in Figure 1A (top) and relative percentages in Figure 1B (bottom). Mean caffeine consumption for the population aged ≥ four years was 135 mg/day. Caffeine consumption increased from 15 mg/day for the youngest age group (four to eight years) to 213 mg/day for the 51–70 years age group and decreased thereafter (138 mg/day among older adults). Overall, the largest contributors to dietary caffeine were coffee (64%), tea (18%), and caffeinated sodas (15%). Energy drinks accounted for 2% of caffeine (2.7 mg/day) and foods contributed 2.0 mg/day or 1.5% of total caffeine. The major sources of caffeine changed with age. While children consumed little caffeine overall, the most important sources were tea and soda followed by foods and flavored milk. Coffee, followed by tea, was the principal source of caffeine for adults.

### 3.2. Fourteen-Year Trends in Caffeine Consumption

**Figure 2:**
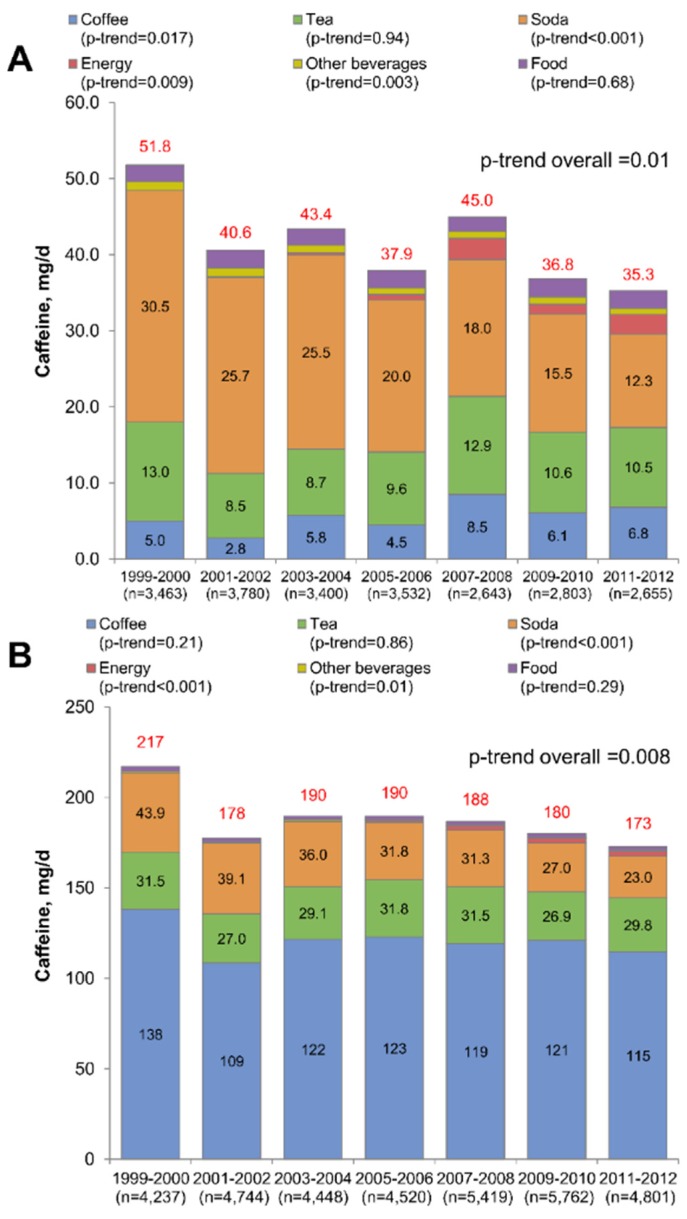
Trends in caffeine consumption overall and by source, stratified by age group. Data are for NHANES 1999–2012. Data are for (**A**) children; (**B**) Adults.

Figure 2 displays fourteen-year trends in caffeine consumption overall, and by food/beverage source, for both children (Figure 2A) and adults (Figure 2B). Among both children and adults, caffeine intakes declined from 175 mg/day in 1999–2000 to 142 mg/day in 2011–2012, largely driven by a drop in caffeine from soda (41 mg/day to 21 mg/day). Among children overall, there was evidence of a decreasing trend, with average caffeine intakes decreasing from 51.8 mg/day in 1999–2000 to 35.3 mg/day in 2011–2012 (*p*-trend = 0.01). There was evidence of a modest increase in caffeine from coffee (increasing from 5.0 mg/day to 6.8 mg/day, *p*-trend = 0.017), and no change in caffeine from tea. Soda was by far the majority source of caffeine for children, but caffeine from soda decreased from 30.5 mg/day to 12.3 mg/day (*p*–trend < 0.001). Caffeine from energy drinks increase significantly from none in 1999–2000, to 0.24 mg/day in 2003–2004, and 2.3 mg/day in 2011–2012. When considering all sources of caffeine other than soda, caffeine consumption changed only modestly from 21.3 mg/day to 23.0 mg/day (*p*-trend = 0.10). Caffeine from consumption of soda and energy drinks combined among children declined (*p*-trend < 0.001), suggesting that the decline in caffeine from soda was not being replaced with caffeine from energy drinks. For adults, we also observed a decrease in caffeine intakes, from 217 mg/day to 173 mg/day (*p*-trend = 0.008). There was no evidence of an increase in caffeine from coffee or tea. Similar to children, a marked decrease in caffeine from soda was observed (from 43.9 mg/day to 23.0 mg/day, *p*-trend < 0.001). There was no evidence that caffeine from all sources, excluding soda, changed over the 14-year study period (*p*-trend = 0.82), among adults, indicating that the observed decline in caffeine consumption could be attributed to declines in caffeinated soda consumption. Among adults, caffeine from energy drinks increased significantly from zero in 1999–2000, to 0.31 mg/day in 2003–2004, and 2.9 mg/day in 2011–2012. When considering soda and energy drinks together, there was still evidence of a decline in caffeine consumption (*p*-trend < 0.001).

Due to concerns that the observed trends among adults could be due to extreme caffeine intakes, a secondary analysis evaluated mean caffeine consumption overall and by beverage source after dropping 293 individuals who reported consuming more than 1000 mg/day (corresponding roughly to 10.5 cups of brewed coffee). After applying this exclusion, there was a modest attenuation of the trend, though there was still evidence of a decrease in total caffeine consumption (*p*-trend < 0.021).

An additional analysis examined trends in caffeine consumption from coffee, tea, and soda among consumers of each of these beverages respectively (data not shown). This analysis was conducted to test whether decreases in caffeine from a given source could be explained entirely by a decrease in the proportion of individuals consuming each product. For each beverage type there was evidence of a decrease in caffeine from each source (*p*-trend < 0.009 for each). These results suggest that consumers of each beverage type are either drinking less of that beverage or shifting towards decaffeinated or lower-caffeinated forms of each beverage.

Sensitivity analyses were conducted to determine if demographic changes, specifically population aging and an increasing number of non-white residents could explain the observed trends. Trends were evaluated after adjusting for age group and race/ethnicity. After adjusting for age, the strength of the trend in caffeine intake became stronger (*p*-trend = 0.001); after adjustment for age and race/ethnicity, the association remained significant, but was weaker than the age-adjusted analysis (*p*-trend = 0.011). The age- and race/ethnicity adjusted trend was very similar to the unadjusted trend.

### 3.3. Fourteen-Year Trends in Caffeine Consumption by Age Group

**Figure 3:**
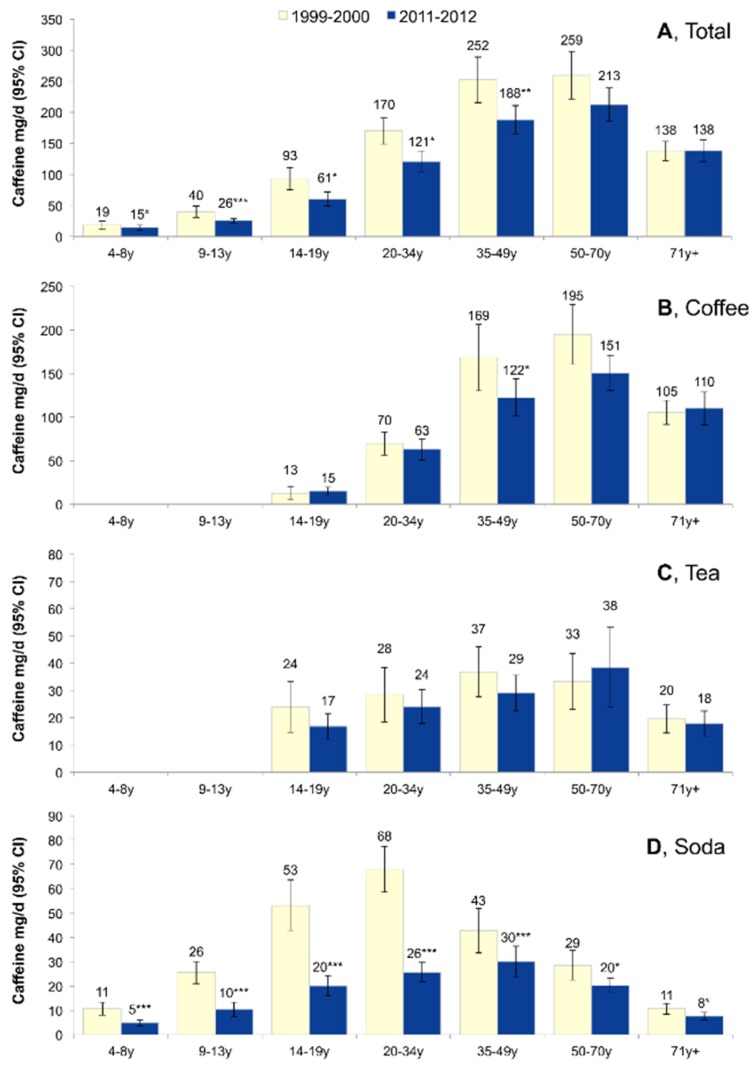
Trends in caffeine consumption stratified by age group and beverage type. Data are for NHANES 1999–2000 and for NHANES 2011–2012. Data are for (**A**) total caffeine; (**B**) Coffee; (**C**) Tea; (**D**) Soda. The *p*-interaction for the age by year interaction was *p* = 0.016, *p* = 0.12, *p* = 0.58, and *p* < 0.001 for total caffeine, coffee, tea, and soda, respectively. Error bars are 95% confidence intervals.

Time trends in caffeine intakes from 1999–2001 to 2011–2012 by age group and beverage type are summarized in Figure 3. There was evidence that the trend differed by age group (*p*-interaction = 0.016). For children and younger adults there was evidence of a decline in total caffeine consumption. There was no evidence of a trend in diminished total caffeine for older adults (*p*-trend = 0.30 for those 50–70 years and *p*-trend = 0.91 for those ≥ 71 years). There was no evidence of an interaction by age group for the trend in caffeine from coffee or tea (*p*-interaction = 0.12 and *p*-interaction = 0.59, respectively). The trend in caffeine from soda did differ by age group (*p*-interaction < 0.001), and declines were observed for all age groups, with the strongest being observed for adolescents age 14–19 years and young adults 20–34 years, groups that also had the highest baseline caffeine intakes from soda.

### 3.4. Trends in Location of Origin for Caffeine, 2003–2012

**Figure 4:**
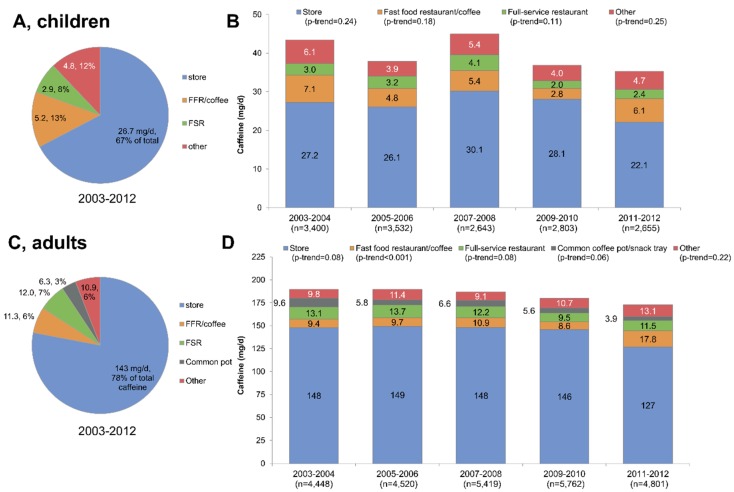
Trends in caffeine consumption by purchase location of origin, shown separately for children (**A**,**B**) and adults (**C**,**D**). Data are for NHANES 2003–2012. FFR stands for fast food restaurant; FSR stands for full-service restaurant.

Time trends in caffeine consumption by purchase location of origin are summarized in Figure 4, separately for children and adults. First, total caffeine consumption declined between 2003–2004 and 2011–2012 for both children and adults. Second, as shown in Figure 4A,C, the bulk of caffeine consumed (67% for children and 78% for adults) came from store-bought coffee, tea, soda, and other foods/beverages. Whereas a downward trend in store-bought caffeine was observed, a sharp increase in 2011–2012 was observed for caffeine from fast food restaurants (Figure 4D).

**Figure 5:**
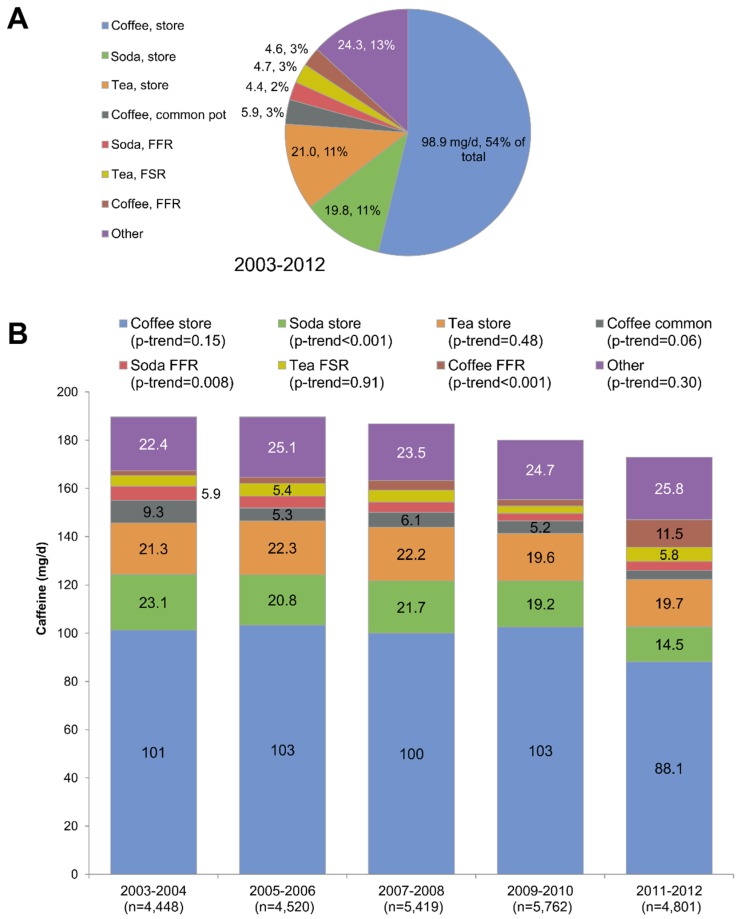
Trends in caffeine consumption by purchase location of origin and beverage type for population aged > 4 years. Data are for the period 2003–2012 (**A**) and shown separately for each NHANES cycle (**B**). FFR stands for fast food restaurant; FSR stands for full-service restaurant.

Figure 5 shows time trends for caffeine consumption by adults from 2003 to 2012 by location of origin and beverage type. As shown in Figure 5A, 99 mg/day of caffeine (or 54% of total) came from store-bought coffee. Another 21 mg/day (11%) came from store-bought tea and 20 mg/day (11%) from store-bought sodas (11%), followed by coffee from a common pot and from fast food restaurants or coffee shops. Figure 5B, illustrating time trends, shows a decline in caffeine from store bought soda and an increase in caffeine from FFR and coffee shops. Both trends were significant.

## 4. Discussion

The present analyses of current caffeine consumption patterns and time trends between 1999 and 2012 add to the growing literature on the dietary sources of caffeine in the US [3,11,15]. The present analyses, based on the most recent 2011–2012 NHANES, confirm the downward trend in caffeine intakes from caffeine containing beverages and foods. Whereas one recent study based on NHANES 2001–2010 [24], estimated caffeine consumption at 211 mg/day for men and 161 mg/day for women, the present estimates based on the most recent NHANES 2011–2012 data were 196 mg/day for men and 151 mg/day for women. For children and adolescents (ages 4–19 years), caffeine intakes were estimated at 35 mg/day. The bulk of caffeine was supplied by store-bought beverages, particularly coffee, tea and soda. The observed decline in caffeine intakes was driven largely by a decline in soda consumption, particularly by teenagers and young adults.

Previous consumption estimates, based on 37,603 persons aged ≥ two years in the Kantar Worldpanel 2010–2011 [3] were 164 mg/day caffeine, with 64% coming from coffee and 17% each from tea and sodas. Energy drinks accounted for 2.0% of total caffeine. The present estimates for persons > four years in 2011–2012 were 135 mg/day caffeine, with 66% coming from coffee, 18.5% from tea, 15.6% from soda, and 2.0% from energy drinks. The Kantar Worldpanel was based on seven day diaries for 37,602 consumers of caffeine (out of 42,851) collected from October 2010 through September 2011. There was good agreement between the present data and the Kantar Worldpanel, even with substantial differences in the methodologies. Our US-based estimates are also consistent with reports from elsewhere. For example, in Germany coffee and tea were observed to be the primary sources of caffeine among adults, while tea and cola were the predominant source of caffeine among those 10–19 years [25]. However, given the comparatively high consumption of soda, including both sugar-sweetened and low-calorie sweetened varieties, soda contributed greater amounts of caffeine in the US.

Analyses of NHANES consumption trends by age showed that total caffeine consumption increased several-fold across the lifespan, consistent with other reports [3]. Whereas soda was the principal source of caffeine for children and adolescents, coffee was the major source among those aged > 20 years. The absolute amounts of caffeine need to be considered: 10 mg of caffeine from soda provided 38% of a total intake of 26 mg in the 9–13 years age group—but 20 mg of caffeine from soda provided only 9% of total intake of 213 mg in the 51–70 years age group.

Analyses of consumption patterns confirmed that the proportion of beverage consumers varied by both age and beverage type. Whereas the percentages of coffee and tea drinkers increased with age, soda consumption peaked in young adult life and then declined. In previous studies, a large proportion of children < 19 consumed some caffeine on a given day (71%–73%) [10,15]. Peak frequency of energy drink consumption (10.3% in the Kantar data) was observed among teenagers [3]. The percentage of respondents consuming soda ranged from 40% to 50% depending on age. Between 10% and 20% consumed tea, and less than 10% consumed coffee or energy drinks.

The present analyses of time trends across multiple NHANES cycles were consistent with past reports. First, coffee and tea now represent a greater proportion of caffeine intakes as soda consumption has declined across all age groups. Similarly, Branum [11] showed that mean caffeine intakes have been stable but declined among young children. As the contribution of soda to caffeine consumption declines, the relative contribution of coffee and to a lesser extent energy drinks has increased. Because both low-calorie and sugar-sweetened sodas may contain caffeine, trends in both sugar-sweetened beverages and low-calorie sweetened beverages may influence intake. The decline in sugar-sweetened soda consumption in the US is well-documented, and there is some evidence of increases in consumption of low-calorie sweetened beverages [26].

Other studies have come to similar conclusions. Recent data on caffeine intakes and caffeinated food and beverages intakes were presented in a FDA report with data from a consumer panel database and other surveys [7]. An earlier study by Frary [27], based on the Continuing Survey of Food Intakes by Individuals (CSFII), also estimated caffeine consumption from soda, tea and coffee, leading to similar conclusions. Kit *et al.* [14] reported that the consumption of soda significantly declined, but the consumption of energy drinks has increased. These studies add to data collected in the late 1980s and early 1990s [1].

The 2015 Dietary Guidelines for Americans Advisory Committee concluded that among no age group did caffeine intakes exceed safe levels [8]. However, they also noted the lack of consensus regarding safe levels among children and adolescents [8]. Therefore, ongoing and context-specific surveillance of caffeine intakes among children/adolescents should be a priority area for dietary surveillance. Understanding the beverage/food sources of caffeine is essential, but understanding the source of caffeine, as done here, is also critical. Identifying trends by food source (e.g., store, fast food/coffee shop) may have implications for the implementation of interventions should caffeine intakes become a concern.

A number of limitations should be noted. First, like any dietary study, some foods/beverages may be under-reported or portion sizes estimated with error. The under-reporting of some beverages, namely sugar-sweetened beverages, might result in under-estimates of caffeine consumption. Second, for each single food/beverage item reported only a single estimate of caffeine content was used. The dietary database used here contains more than 7000 foods/beverages with numerous options that differentiate decaffeinated and caffeinated beverages. For example, the database includes more than 50 varieties of coffee/coffee drinks, 30 types of tea and numerous caffeinated and decaffeinated versions of soda. Variability in caffeine content may lead to error; this is particularly a concern for brewed coffee and hot tea [25]. Lastly, this analysis focused on dietary sources of caffeine, therefore caffeine from supplements were not included. Despite these limitations, the current data are the best suited to examine US trends in caffeine consumption in a large and nationally representative sample.

## 5. Conclusions

The present study used nationally representative NHANES 1999–2012 data to provide the first analysis of trends in population level caffeine intakes by age group, beverage type, and purchase location of origin. Caffeine intakes in the US remain driven by the consumption of store bought coffee and tea, followed by store-bought carbonated sodas [3]. The consumption of caffeine from all sources by children remains low.

## Figures and Tables

**Table 1:** Average caffeine consumption by race/ethnicity, family income, educational attainment and employment status among children/adolescents and adults, 2011–2012.

	Age 4–19 Years (*n* = 2655)			Age ≥ 20 Years (*n* = 4801)		
	*n*	Mean	95% CI	*n*	Mean	95% CI
Total	2655	35	31, 39	4801	173	154, 192
Gender						
Male (ref)	1357	36	31, 43	2394	196	165, 226
Female	1298	34	28, 40	2407	151 ***	138, 164
Race/ethnicity ^a^						
Non-Hispanic White (ref)	605	47	38, 55	1842	205	185, 225
Non-Hispanic Black	784	20 ***	15, 25	1274	78 ***	65, 92
Non-Hispanic Asian	317	26 ***	18, 34	610	108 ***	93, 122
Mexican American	504	23 ***	17, 29	467	117 ***	96, 139
Other Hispanic	303	24 ***	20, 29	465	110 ***	86, 135
Family income-to-poverty ratio						
<1.3	1167	36	26, 47	1564	146 *	117, 176
1.3–1.849	335	38	26, 51	594	164	138, 191
1.85–2.99	350	31	22, 40	702	186	159, 214
≥3.0 (ref)	617	33	27, 40	1574	189	165, 213
Educational attainment ^b^						
<HS				1044	171	131, 212
High school				904	185	150, 221
Some college				1210	198 *	176, 220
≥College graduate (ref)				1158	173	152, 194
Employment status						
Employed (ref)				2565	186	171, 200
Not employed				2235	158 *	131, 184

^a^ Data for other race/mixed race is not presented due to small numbers. These individuals are included in all other analyses; ^b^ Limited to adults age ≥ 25 years since adults 20–24 years have often not completed their education; *** *p* < 0.001, ** 0.001 < *p* < 0.01 and * 0.01 < *p* < 0.05 from pairwise survey-weighted *t*-test comparing mean to the reference group (identified in parentheses).

## Abbreviations

The following abbreviations are used in this manuscript:

NHANES: National Health and Nutrition Examination Survey
USDA: United States Department of Agriculture
FNDDS: Food and Nutrient Database for Dietary Studies
